# Assessing impact of MALDI mass spectroscopy on reducing directed antibiotic coverage time for Gram-negative organisms

**DOI:** 10.1371/journal.pone.0228935

**Published:** 2020-02-26

**Authors:** Calvin Ka-Fung Lo, Dominik Mertz, Debbie Yamamura, Mark Loeb

**Affiliations:** 1 Hamilton Regional Laboratory Program, Hamilton, Ontario, Canada; 2 Department of Medicine, University of British Columbia, Vancouver, British Columbia, Canada; 3 Department of Medicine, McMaster University, Hamilton, Ontario, Canada; 4 Department of Health Research Methods, Evidence and Impact, McMaster University, Hamilton, Ontario, Canada; 5 Michael G. DeGroote Institute for Infectious Diseases Research, McMaster University, Hamilton, Ontario, Canada; Fisheries and Oceans Canada, CANADA

## Abstract

The objective of this study was to assess whether use of matrix assisted laser desorption ionization-time of flight (MALDI-TOF), through improvements in identification time, reduces time to directed antibiotic coverage. We therefore conducted a retrospective review of 377 blood cultures from hospitalized patients with gram negative bacteremia that underwent testing by MALDI-TOF compared to standard identification methods (VITEK 2) for blood cultures from January 2016 to December 2017. We found that MALDI significantly reduced time between blood culture collection to reach pathogen identification and was associated with a significantly reduced time to initiate more specific therapy, with a mean difference of 16.37 hours, 95% CI 10.05 to 22.69 (mean time 50.34 hours (+/- 21.21) vs VITEK: 66.71 hrs (+/- 27.12), p<0.001 as well as a reduced time to discontinue previous therapy (p = 0.004). In conclusion, in reducing time to identification of gram negative bacteremia, MALDI-TOF led to improvements in antibiotic coverage.

## Introduction

Matrix-assisted laser desorption ionization time-of-flight (MALDI-ToF or MALDI), through mass spectroscopy, can rapidly identify proteins in bacteria [1]. Compared to traditional identification methods which may take 4 to 18 hours, MALDI averages 45 to 90 minutes to identify isolates from the point of inoculation [2]. MALDI has high accuracy in identifying bacterial species, estimated to be up to 97.7% for *Enterobacteraciae* and 75% for non-fermentative Gram-negative bacilli when used directly on positive blood bottles [3]. In a study of 203 positive blood cultures, identification at 6 hours (93% of the cultures) was 94% accurate by mass spectroscopy, with essential and categorical agreement for gram negative bacilli being 99.1 and 99% respectively [4]. Common clinical isolates can be identified within 5 hours and susceptibility reported within 12 hours [5].

Implementation of more rapid diagnostic technologies may also improve the likelihood of positive clinical outcomes. For example, one study demonstrated significant reduction in infection-related length of stay since implementing MALDI (10.5 days to 8.3 days, p = 0.006) and median antibiotic costs per course ($23.90 to $14.60, p = 0.002) [6]. This study was limited however by being a single-centre study with only pediatric cases [6]. Moreover, limited microbiology staffing and daytime administration at this site led to batching and processing samples twice daily (i.e., 8 am to 4 pm), which led to the inability to perform antibiotic stewardship interventions in real-time if specimens were identified in post-daytime hours [6].

Whether MALDI leads to improved antibiotic coverage is uncertain as only two studies have addressed this question. One study of 253 culture episodes reported an 11.3% increase in appropriate antibiotic use [7]. The other study reported that of 202 cultures, MALDI-TOF lead to a 14.3% increase in therapy modification beyond changes made on the basis of conventional Gram stain [8]. Both studies were limited by small sample sizes [7, 8], conducted in regions with low levels of antimicrobial resistance [7, 8] and had sub-optimal designs (single arm) [8].

The purpose of this study was to compare time to identification of the pathogen comparing MALDI versus traditional blood cultures/VITEK 2 (termed “standard of care” throughout the study) and the impact on antimicrobial management during a time period where a mix of these two methods were used.

## Materials and methods

The study protocol including definition of eligibility criteria, study design, data collection, and how the data will be analyzed was established prior to data collection. The Hamilton Integrated Research Ethics Board (HiREB) reviewed and granted approval for our study, most recently renewed in March 2019 (Study Number: 4521). Data was collected through retrospective patient health records, specifically patients with gram negative bacteremia admitted to two acute care tertiary centres in Hamilton, Ontario, Canada (Hamilton General and Juravinski). Date ranges for our retrospective records were from January 1, 2016 to December 31, 2017. Patients were anonymized during collection and no identifiers were collected for purposes of our study. Please refer to Eligibility criteria and Data Collection below for additional details.

### Eligibility criteria

Patients with gram negative bacteremia who had been admitted to two acute care tertiary centers in Hamilton, ON, Canada (i.e., Hamilton General and Juravinski) from January 1, 2016 to December 31, 2017 were assessed. During this period, both standard identification methods and MALDI-TOF were used. Culture with the following clinically significant Gram-negative species were included: *Acinetobacter baumannii* (and species), *Enterobacter cloacae* complex, *Klebsiella pneumoniae*, *Pseudomonas aeruginosa*, *Serratia marcescens and Stenotrophomonas maltophilia*. Due to the high prevalence of *Escherichia coli* (*E*. *coli*) positive cultures, our decision was not to include *E*. *coli* in order to prevent unequal representation of pathogen specimens when interpreting outcome measures.

Only a patient’s first admission during the calendar year was eligible in order to prevent duplicate entries during analysis. If a patient had several specimen entries in the same calendar year, we extracted only the first specimen entry during admission. If a patient had a poly-microbial infection, we only recorded the primary Gram-negative pathogen of interest. Antibiotic susceptibility results were recorded for each respective culture.

### Data collection

Data was extracted from the electronic health record system using a standardized data form. The date and time was collected for the following: blood culture collection, receipt of the specimen at the microbiology laboratory; verbal reporting of the critical gram stain, electronic reporting of the identification and susceptibility results. The identification method and organism identification to species level was recorded. We documented start and end-dates of antibiotic prescriptions during patient hospitalization from one week prior to the date of culture collection up to three days after susceptibility results. Based on microbiologic classifications, cultures were also classified as Enterobacteriaceae (*Enterobacter cloacae*, *Klebsiella pneumoniae* and *Serratia marcescens*) or non-fermenters *(Pseudomonas aeruginosa*, *Acinetobacter* species, *Stenotrophomonas maltophilia*) for secondary analysis.

### Data analysis

The primary outcome for this study was reduction in time to directed coverage of antibiotics based on identification. Secondary outcomes were specific changes to empiric antibiotics used, and time until pathogen identification and antibiotic susceptibilities.

**Fig 1:**
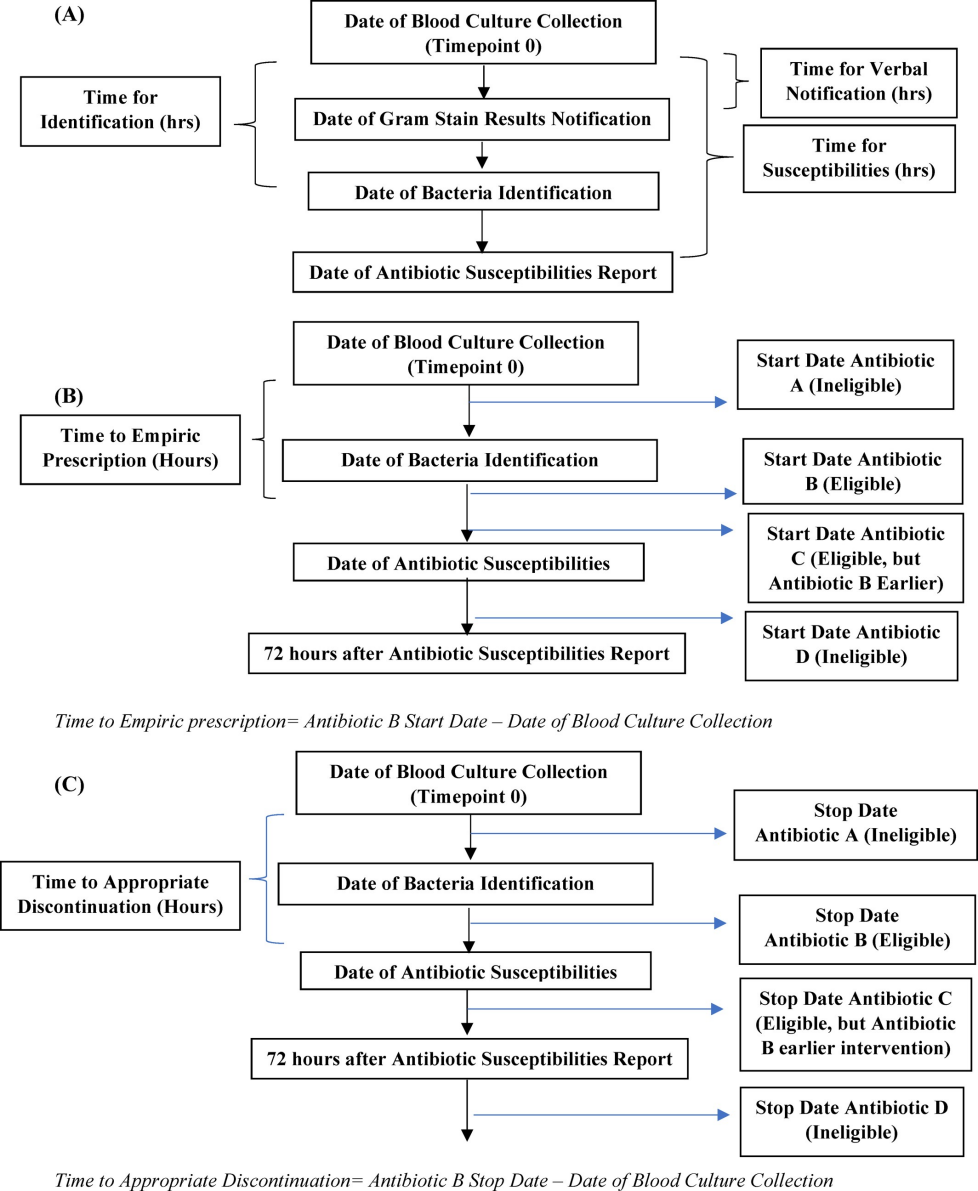
Standard Timepoints For Study Collection, Screening Culture Eligibility based on Antibiotic Prescription and Discontinuation Dates. **(A**) Based on Timepoint 0 as start point, we collected time elapsed from each subsequent date (Gram Stain Verbal Report, bacterial identification and antibiotic susceptibilities). (**B)** Cultures associated with ≥ 1 antibiotic prescription between date of bacterial identification and date of antibiotic susceptibilities were eligible for prescription analysis. As shown in B, culture had two associated antibiotics that met criteria (Antibiotic B and C). Only timeline for Antibiotic B would have been considered, since it had earliest start time. Subsequently, difference between start date for antibiotic B and date of blood culture collection will be calculated. (**C)** Cultures associated with ≥ 1 antibiotic prescription discontinued between “date of bacterial identification” and “72 hours post antibiotic susceptibility” were eligible for discontinuation time analysis. Here, antibiotics A and D would be ineligible, only antibiotic B and C met the criteria. We used earliest eligible antibiotic discontinued date (i.e., antibiotic B) and calculated difference between antibiotic B stop date with respect to date of blood culture collection.

Screening for eligible cultures and data extraction were performed first. Specific timepoints for the analyses were then assessed. The starting timepoint for our analyses was the time when the blood culture was collected. Time to pathogen identification and susceptibilities were then calculated using “Time of Identification” and “Time of Susceptibility” with respect to the time of collection, as illustrated in Fig 1A. To further characterize culture workflow, we also determined “Time for Gram Stain Result Notification” and determined the time elapsed since blood culture collection.

We also reviewed each patient’s antibiotic prescriptions during their hospitalization. Patients were eligible for the prescription analysis if there was at least one antibiotic listed that started between the time of identification of the pathogen and the report of susceptibilities. If there were more than one eligible antibiotic, we examined the earliest prescription start time among the eligible antibiotics and determined time difference from date of culture collection, as illustrated in Fig 1B. Cultures where identification and susceptibilities were electronically reported at the same time were excluded from this prescription analysis given there would be no existing time gap for antibiotics to be initiated prior to susceptibilities being reported. Antibiotics initiated before bacterial identification were not considered, because the method for culture identification (MALDI or VITEK 2) would not have played a role and therefore would not have been relevant to decision making about prescribing. Antibiotics started after susceptibility results were also not considered for analysis because over the study period, timing of susceptibility results was the same whether MALDI or VITEK 2 was used. In essence, that would not provide much insight whether testing modality was a major factor in expediting initiation of antibiotic therapy.

We used a similar approach for discontinuing antibiotic coverage except that we considered the prescription end dates. Patients were eligible if they had at least one antibiotic that was discontinued between the time of bacterial identification to 72 hours after the date of the susceptibility report. Antibiotics that were discontinued prior to pathogen identification were excluded, as were antibiotics stopped >72 hours after susceptibility results were reported. We collected the earliest eligible discontinuation date and determined the difference from time of culture collection (Fig 1C).

In addition, we also evaluated the appropriateness of antibiotic stewardship response post-identification. We reviewed the earliest start of antibiotic prescriptions and defined them as appropriate or inappropriate based on the identification of the gram-negative bacteria and the antibiotic selection. We used a similar approach for discontinuation of antibiotics for each culture, defining discontinuations that were clearly appropriate for each culture. Antibiotic therapies that could not be clearly classified as either appropriate or inappropriate were removed from this additional analysis. Definitions of appropriate treatment were based on knowledge of local resistant patterns. These included, for *Acinetobacter baumannii*, use of ceftazidime, piperacillin-tazobactam, fluoroquinolone/aminoglycoside in combination with another antibiotic; for *Enterobacter cloacae*, use of a carbapenem or ciprofloxacin; for *Klebsiella pneumoniae*, *use of* piperacillin-tazobactam, ceftriaxone, ciprofloxacin/levofloxacin, carbapenems; for *Pseudomonas aeruginosa*, use of ceftazidime, piperacillin-tazobactam, meropenem or imipenem, ciprofloxacin, aminoglycoside; for *Serratia marcescens*, ciprofloxacin, ertapenem; and for *Stenotrophomonas maltophilia*, trimethoprim-sulfa, levofloxacin, ciprofloxacin, ceftazidime.

We assessed the impact of the MALDI on all samples and also compared outcomes between Enterobacteriaceae to non-fermenters.

We used the student t-test to compare means and chi-square test to compare proportions. All statistical analyses were conducted using SPSS/PASW Version 18 (SPSS Inc., Chicago, IL). The threshold for significance is 0.05.

## Results

After screening for duplicate or multiple entries, 16 patients had more than one admission in the same calendar year and 56 patients had more than one set of cultures taken on separate dates. A total of 381 patient cultures were reviewed, of which 377 met our eligibility criteria. Three cultures underwent phenotypic assessment (e.g., morphology, oxidase) to identify organism and one did not grow any of the six Gram-negative organisms of interest for our study. Among eligible cases, there were 170 female patients (45.1%) in the study and the median age was 65.0 years (interquartile range 52.0–76.0 years). 48.5% of the cultures (183/377) were processed through MALDI for identification.

Of the 377 patient cultures, there were 145 (38.5%) that were *Klebsiella pneumoniae*, 102 (27.1%) *Pseudomonas aeruginosa*, and 51 (13.5%) *Enterobacter cloacae* (Table 1). Based on our classification criteria (S2 File), 61.5% of the cultures were Enterobacteriaceae.

**Table 1:** Frequencies of gram-negative pathogens among patient cultures (n = 377) and percentage of cultures per testing modality.

Gram-Negative Pathogen Name	Frequency (%)	Number of MALDI cultures (%)	Number of VITEK cultures (%)
***Klebsiella pneumoniae***	145 (38.46)	69 (47.59%)	76 (52.41%)
***Pseudomonas aeruginosa***	102 (27.06)	38 (37.25%)	64 (62.75%)
***Enterobacter cloacae* complex**	51 (13.53)	41 (80.39%)	10 (19.61%)
***Serratia marcescens***	36 (9.55)	13 (36.11%)	23 (63.89%)
***Acinetobacter* species**	22 (5.84)	11 (50%)	11 (50%)
***Stenotrophomonas maltophilia***	21 (5.57)	11 (52.38%)	10 (47.62%)

Most cultures were flagged positive and subsequently reported during shift periods 0700 to 2300, 61.0% (105/172) for the MALDI group and 70.7% (130/184) for VITEK.

### Antibiotic frequencies

613 antibiotics were prescribed for the 377 patients. The total number of antibiotics prescribed for each positive blood culture ranged from 0 to 5 antibiotics, the median number was 2 (with < 20% of blood cultures having > 2 antibiotics prescribed). Among the 36 cultures with no antibiotics prescribed, 19 were in patients who died prior to culture identification results being generated and 2 after susceptibility reports were generated. The five most frequently prescribed antibiotics were ciprofloxacin (169/613, 27.6%), piperacillin/tazobactam (156/613, 25.4%), meropenem (83/613, 13.5%), ceftriaxone (61/613, 9.9%) and ertapenem (45/613, 7.34%).

### Clinical outcomes for specimen reporting, identification and susceptibilities

For 352 blood cultures, there was a significantly shorter time from blood culture collection to reporting positive Gram stain cultures for the MALDI blood culture group compared to the group where standard methods were used (18.42 hours (± 7.17) versus 20.29 hours (± 9.09), respectively (mean difference 1.87 hours, 95% CI 0.14 to 3.59, p = 0.034). MALDI had a reduced mean identification time of 34.58 hours (± 14.60) compared to 48.91 hours (± 16.47) for standard methods, mean difference 14.33 hours, 95% CI 11.15–17.51, p<0.001. Mean identification time from positive gram stain also remained significantly different between testing modalities (MALDI 15.59 hours ± 11.80 vs. VITEK 28.27 hours ± 14.48 respectively; mean difference 12.68 hours, 95% CI 9.91 to 15.45, p <0.001). 365 blood cultures had valid susceptibility result times, which also showed no significant difference until susceptibilities became available (48.69 hours ± 30.35 vs. 52.33 hours ± 22.62 respectively; mean difference 3.63 hours, 95% CI -1.84 to 9.11, p = 0.193) for MALDI compared to standard methods (Table 2).

**Table 2:** Mean times gram stain verbal reporting, bacterial identification and antibiotic susceptibilities*.

Testing Modality Outcomes	MALDI (number of cultures)	VITEK (Number of Cultures)	Mean Differences in hours (95% CI, p-value)
**Gram Stain Verbal Reporting Time in hours**	18.42 (170)	20.29 (182)	1.87 (0.14 to 3.59, p = 0.034)
**Time for Bacteria Identification- hours (from Blood Culture Collection; from Gram Stain Verbal Report)**	34.58 (180); 15.59 (172)	48.91 (193); 28.27 (183)	14.33 (11.15–17.51, p<0.001); 12.68 (9.91–15.45, p<0.001)
**Time for Antibiotic Susceptibilities- hours**	48.69 (173)	52.33 (192)	3.63 (-1.84 to 9.11, p = 0.193)

*All times are with respect to date of culture collection, unless indicated otherwise. Among 377 blood culture data collected in our study, a total of 184 cultures were processed by MALDI and 194 by VITEK. Cultures with valid Gram-Stain reporting (170 MALDI, 182 VITEK = total 352 cultures), Specimen Identification (180 MALDI, 193 VITEK = 373 cultures) and antibiotic susceptibility times (173 MALDI, 192 VITEK = 365) were respectively used for this analysis.

### Times to prescribe empiric therapy

After screening for cases where antibiotic therapy was prescribed, a total of 228 cultures were analyzed with 57% (129/228) of cultures processed through MALDI. Cultures processed through MALDI had a significantly reduced time to initiate post-identification antibiotic therapy, with a mean difference of 16.37 hours, 95% CI 10.05–22.69; 50.34 hours (+/- 21.21) for MALDI versus 66.71 hrs (+/- 27.12) for standard methods, p<0.001) (Table 3).

**Table 3:** Time to empiric prescriptions and discontinuation of empiric therapy- MALDI vs. VITEK*.

Testing Modality Outcomes	MALDI (number of cultures)	VITEK (Number of Cultures)	Mean Differences in hours (95% CI, p-value)
**Time to Empiric Prescription- hours**	50.34 (129)	66.71 (98)	16.37 (10.05 to 22.69, p<0.001)
**Time to Empiric Therapy Discontinuation–hours**	58.21 (132)	68.39 (120)	10.19 (3.32 to 17.06, p = 0.04)

*All times are with respect to date of culture collection, unless indicated otherwise.

### Time to discontinue empiric therapy

There were 252 cultures analyzed for time to discontinuation of antibiotics. Cultures with MALDI processing had a significantly reduced time to discontinuation of empiric antibiotics that were no longer appropriate after knowing the identification of the pathogen. The average time being 58.21 hours (+/- 26.77) vs standard methods: 68.39 hours (+/- 28.61), with a mean difference of 10.19 hours, 95% CI 3.32–17.06, p = 0.004 (**Table 3**).

### Additional analyses: Pathogen species

Significant reductions in prescription time remained for MALDI-processed cultures compared to VITEK 2 when stratifying cultures by Enterobacteraciae (mean difference 18.39, 95% CI 10.63–26.15, p<0.001) or non-fermenters (mean difference 12.62, 95% CI 1.49–23.75, p = 0.027). In terms of discontinuation times for empiric therapies, only Enterobacteriaceae cultures demonstrated significant reduction in time (55.07 hours versus 68.44 hours, mean difference 13.37, 95% CI 5.15–21.59, p = 0.002 (Table 4).

**Table 4:** Secondary analyses for empiric prescription and discontinuation times across MALDI vs. VITEK–enterobacteriaceae vs. Non-fermenters*; appropriateness of therapy changes.

Outcomes		MALDI (number of cultures)	VITEK (number of cultures)	Mean differences in hours (95% CI, p-value)
**Time to empiric prescription—hours**†	**Enterobacteriaceae**	**48.80 (86)**	**67.18 (57)**	**18.39 (10.63–26.15, p<0.001)**
	**Non-Fermenters**	**53.43 (43)**	**66.05 (41)**	**12.62 (1.49 to 23.75, p = 0.027)**
	**Appropriate prescription Starts**	**50.75 hours (100)**	**66.42 hours (81)**	**15.66 hours (8.42–22.91, p<0.001)**
**Time to empiric therapy discontinuation- hours**	**Enterobacteriaceae**	**55.07 (86)**	**68.44 (68)**	**13.37 (5.15–21.59, p = 0.002)**
	Non-Fermenters	64.07 (46)	68.33 (52)	4.26 (-7.96 to 16.48, p = 0.49)
	**Appropriate prescription discontinuations**	53.09 (47)**	**65.00 (47)**	**11.91 hours (1.98–21.84, p = 0.019)**

*Enterobacteriaceae: Enterobacter cloacae complex, Klebisella pneumoniae, Serratia marcescens. Non-fermenters: Pseudomonas aeruginosa, Acinetobacter species, Stenotrophomonas maltophilia

† Empiric Prescription- earliest antibiotic prescribed after the bacteria species was identified (i.e., Date of Bacteria Identification), please see Fig 1B for visual representation

** One of the MALDI Cultures in this Database had appropriate discontinuation but due to lacking a valid start time (i.e., time of blood culture collection), it was removed from analysis hence only 47 cultures in this group.

### Appropriateness of antibiotic changes

Among the 227 cultures, a total of 181 cultures (79.7%) were considered appropriate therapy initiations. More specifically, 100/129 (77.52%) patients with identification through MALDI and 81/98 (81.82%) with standard cultures were appropriate. Similar to our original analysis, cultures processed through MALDI had significantly reduced time for appropriate therapy, with mean difference of 15.66 hours, 95% CI 8.42–22.91. The average time for MALDI cultures was 50.75 hours (+/- 21.69) versus 66.42 hours (+/- 27.70) for standard methods, p<0.001.

For discontinuing prescriptions, only 97 cultures had clear classifications with 95 (97.9%) being clearly appropriate and 2/50 (4%) in the MALDI group classified as inappropriate discontinuations. Among the 95 appropriate discontinuations, 94 had valid blood culture collection times with equal group sizes for each testing modality (i.e., 47 cultures each). Likewise, significant differences persisted between the MALDI and standard cultures’ discontinuation times. MALDI cultures averaged 53.09 hours (+/- 23.09) versus 65.00 hours (+/- 25.33) for standard methods, with a mean difference of 11.91 hours, 95% CI 1.98–21.84 hours, p = 0.019. Please see Table 4 for reference.

## Discussion

In our study, MALDI was found to significantly reduce identification times, from 48.91 to 34.58 hours (mean difference 14.33 hours, 95% CI 11.15 to 17.51, p<0.001). Significant reductions in both time to initiate prescription of directed antibiotic therapy and time to discontinue previous antibiotic therapy were also achieved through use of MALDI. Stratifying cultures by Enterobacteracaie or non-fermenters yielded similar results for prescription times. Only cultures for Enterobacteriaceae demonstrated a significant reduction in time to discontinuing inappropriate antibiotics. Review of antibiotic prescriptions demonstrated a high degree of appropriate therapy based on organism identification by either method but with a significant reduction in time to appropriate antibiotics for identification using MALDI versus standard of care.

Our findings are consistent with previous studies with respect to the value of MALDI-ToF in improve turnaround times [6–8]. A previous study by Vlek done in the Netherlands similarly compared a pre- and post-MALDI period and reported a reduction in identification time by 28.8 hours and an increased proportion of patients on appropriate therapy within 24 hours by 11.3% [7]. However, their focus was comparison between two 2- month periods where only MALDI-TOF was exclusively performed during one of the time periods versus standard care in the other. Given that MALDI-TOF MS (mass spectroscopy) alone does not provide information on susceptibilities, the authors acknowledged that their findings were less applicable in countries with higher levels of antibiotic resistance [7]. Likewise, Clerc et al. conducted a prospective study at a single hospital site (Lausanne, Switzerland) on gram-negative bacteremia, which was similar to our study. It also focused primarily on empirical antibiotic choice but included strictly patients whose positive cultures required an infectious disease consultation [8]. Similarly to Vlek, Clerc also acknowledged their study site had <5% incidence of antibiotic-resistant organisms such as ESBL [8]. Compared to our study, Clerc focused on a single-arm design, assessing the percentage of cases where empiric therapy was modified after Gram-Stain (Step 1) and MALDI-ToF reporting (second step), in essence all cultures being processed through MALDI [8]. Their outcome did not focus on our primary interest: time reduction for prescribing or discontinuing therapy. Lastly, Bhavsar also reported improved clinical outcomes with MALDI confirming significant reduction in identification and susceptibility times, although it also focused on infection-related length of stay (reduction from 10.5 days to 8.3 days, p = 0.006) and median antibiotic costs per course ($23.90 to $14.60, p = 0.002) [6]. This study differs from ours in that it involved only pediatric cases and relied on batching of processed samples due to limited microbiology staff, which limits real-time antibiotic stewardship interventions if specimens were identified in post-daytime hours (i.e., 4 pm-8 am) [6].

The reduction in time to identification with MALDI is primarily due to the rapidity of results compared to standard methods. In addition, performance of off-line tests such as catalase and oxidase are not required and unlike standard methods which require sufficient growth on solid agar prior to performing identification, MALDI-ToF requires a lower inoculum to do testing. Using haze growth or direct from blood culture, will further reduce the time to identification [2]. Hence, this reduced turn-around time will allow physicians to be notified earlier and then treat with directed therapy based on organisms identification, pending susceptibility results.

Strengths of our study included the use of clinically relevant outcomes, a focus on both the initiation and discontinuation of antibiotics, a large sample size, and the exclusion of repeat patient admissions and multiple cultures, thus keeping events in the analysis independent. However, we acknowledge a number of limitations. First, only a primary pathogen was assessed in situations where there were multiple pathogens. Second, approximately 38% of our baseline eligible cultures were deemed ineligible for prescription time analysis due to having antibiotics prescribed prior to the marked identification timepoint. Third, the lack of significant reduction for susceptibilities reporting in our setting is another limitation. This is due to the fact that during our study time period (January 2016-December 2017), there were 129/377 (34.2%) culture reports where time of release of identification results were not released until the susceptibility reports were also generated, essentially these data points were reported at the same time whether these cultures were in MALDI or VITEK 2 group. Fourth, our focus on gram negative bacteria alone, excluding *E*. *coli* cultures, is a limitation. Lastly, we were only able to access pharmacy orders through Patient Care Inquiry within the electronic health record system, which may not perfectly reflect the true time physicians documented their antibiotic orders, potentially suggesting even earlier times for modifying antibiotic therapy than our study findings suggest.

In summary, we found that MALDI, through reduced identification times, leads to clinically valuable optimization of antibiotics through both early initiation and early discontinuation of antibiotics for gram negative bacteremia.

## Supporting information

S1 File Dataset of Blood Cultures and Antibiotics used for study analysis. (XLSX)

S2 File Summary notes—Classification of Enterobacteriaceae vs. Non-fermenter species, Note 2 –Definitions of Appropriate Antibiotic treatment per species. (DOCX)
