# Supplementary material for: Assessing impact of MALDI mass spectroscopy on reducing directed antibiotic coverage time for Gram-negative organisms
Source: PLoS One. 2020 Feb 26;15(2):e0228935. doi: 10.1371/journal.pone.0228935 (PMC7043764; doi:10.1371/journal.pone.0228935)
Supplement: S2 File — (DOCX) [file pone.0228935.s002.docx]

**APPENDIX Note 1:** For classifying cultures, there were six main pathogen groups among our patient culture list. Among the six, three are classified as “Enterobacteriaceae” specifically cultures containing *Enterobacter cloacae, Klebsiella pneumoniae* and *Serratia marcescens*. The remaining culture specimens (i.e., *Pseudomonas aeruginosa, Acinetobacter baumannii, Stenotrophomonas maltophi*) were noted as “non-fermenters” during our secondary analysis. Classification criteria were based on Jawetz, Melnick & Adelberg’s Medical Microbiology 28^th^ edition (Riedel et al., 2019).

**APPENDIX NOTE 2:** Definitions of Appropriate Treatment. Based on ML’s expert opinion, A.baumannii – ceftazidime, Pip-Tazo, fluoroquinolone/aminoglycoside in combination with another antibiotic. E. cloacae: carbapenem or ciprofloxacin. K. pneumoniae: Pip-Tazo, ceftriaxone, ciprofloxacin/levofloxacin, carbapenems). P. aeruginosa: refer to typical anti-pseudomonal agents), S. marcescens: ciprofloxacin, ertapenem. S. maltophi – Septra, levofloxacin, ciprofloxacin, ceftazidime.
